# Multiple Odontogenic Keratocysts as the Initial Manifestation of Gorlin Syndrome in a Child: A Case Report With Conservative Management and Diagnostic Considerations

**DOI:** 10.1155/crid/1332643

**Published:** 2026-10-10

**Authors:** V. Anguera-Ortega, P. Cahuana-Bartra, L. Brunet-Llobet, Y. González-Chópite, E. I. Mashala, J. Miranda-Rius

**Affiliations:** ^1^ Department of Dentistry Hospital Sant Joan de Déu University of Barcelona Barcelona Spain ub.edu; ^2^ Department of Odontostomatology Faculty of Medicine and Health Sciences University of Barcelona Barcelona Spain ub.edu; ^3^ Hospital Dentistry and Periodontal Medicine Research Group Institut de Recerca Sant Joan de Déu (IRSJD) Barcelona Spain sjdhospitalbarcelona.org; ^4^ Department of Orthopedic Surgery and Traumatology Mount Meru Regional Referral Hospital Arusha Tanzania moh.go.tz

**Keywords:** case report, decompression, Gorlin syndrome, nevoid basal cell carcinoma syndrome, odontogenic keratocyst, pediatric dentistry, PTCH1

## Abstract

Multiple odontogenic keratocysts are one of the major diagnostic criteria of Gorlin syndrome and may represent an early manifestation of the disorder in pediatric patients. This report describes the diagnostic process and management of an 11‐year‐old boy who presented with multiple mandibular cystic lesions associated with displacement of developing permanent teeth. Clinical and radiographic examination, including cone‐beam computed tomography, revealed several well‐defined radiolucent lesions suggestive of odontogenic keratocysts. A conservative treatment approach was adopted, consisting of decompression of the largest lesion and enucleation of smaller lesions, with the aim of preserving developing dentition and reducing treatment‐related morbidity. Histopathological examination confirmed the diagnosis of odontogenic keratocysts. Genetic evaluation by Sanger sequencing identified a heterozygous de novo PTCH1 variant, NM_000264.5(PTCH1):c.1340T>A (p.Leu447∗), confirming the diagnosis of Gorlin syndrome. After 18 months of radiographic surveillance and 30 months of clinical monitoring, progressive bone regeneration, reduction in lesion size, favorable spontaneous repositioning of the affected teeth, and no evidence of recurrence were observed. This case highlights the diagnostic value of multiple odontogenic keratocysts as an early manifestation of Gorlin syndrome and supports conservative management and multidisciplinary follow‐up in growing patients.

## 1. Introduction

Gorlin syndrome (GS), also known as nevoid basal cell carcinoma syndrome (NBCCS), is a rare autosomal dominant disorder with an estimated prevalence ranging from 1/31,000 to 1/164,000 individuals [1]. The syndrome is characterized by multisystem involvement affecting the skin, skeleton, central nervous system, eyes, and craniofacial structures, as well as odontogenic manifestations such as odontogenic keratocysts (OKCs), which constitute one of its major diagnostic criteria [1–3].

Most cases are associated with pathogenic variants in the *PTCH1* gene, a tumor suppressor involved in the Hedgehog signaling pathway that plays a crucial role in embryonic development and cellular growth regulation [2, 3]. Loss‐of‐function variants result in dysregulated cellular proliferation and increased susceptibility to the development of basal cell carcinomas and other characteristic manifestations of the syndrome [2–4].

The diagnostic criteria for GS have evolved substantially since the proposals by Evans et al. and Kimonis et al. [5, 6]. Subsequently, in 2011, the first international consensus of the BCNS Colloquium Group was published, which incorporated molecular analysis as part of the diagnostic confirmation for the first time. A diagnosis is established when two major clinical criteria are met, or one major and two minor criteria, or one major criterion plus molecular confirmation (Table 1) [7].

**Table 1:** Diagnostic criteria for Gorlin–Goltz syndrome (2011) [7].

Major criteria	Minor criteria
BCC prior to 20 years old or excessive numbers of BCCs out of proportion to prior sun exposure and skin type	Rib anomalies
Odontogenic keratocyst of the jaw prior to 20 years of age	Other specific skeletal malformations and radiologic changes (i.e., vertebral anomalies, kyphoscoliosis, short fourth metacarpals, postaxial polydactyly)
Palmar or plantar pitting (three or more)	Macrocephaly
Lamellar calcification of the falx cerebri (< 20 years)	Cleft/lip palate
Medulloblastoma, typically desmoplastic	Ovarian/cardiac fibroma
First degree relative with BCNS	Lymphomesenteric cysts
	Ocular abnormalities (i.e., strabismus, hypertelorism, congenital cataracts, glaucoma, coloboma)

Abbreviations: BCC, basal cell carcinoma; BCNS, basal cell nevus syndrome (Gorlin–Goltz syndrome).

OKCs associated with GS frequently develop during childhood or adolescence and may represent one of the earliest clinically detectable manifestations of the syndrome [4]. Compared with sporadic lesions, syndromic OKCs tend to occur at a younger age, are more frequently multiple, and show a greater tendency for recurrence [4]. Early recognition of these lesions is therefore essential, as timely diagnosis facilitates multidisciplinary management, implementation of surveillance protocols, and early detection of other potentially serious manifestations of the disorder [7].

Although OKCs are a well‐recognized manifestation of GS, they may represent the earliest clinically detectable sign of the disorder during childhood. Early recognition is important because it enables timely multidisciplinary assessment, genetic evaluation, and long‐term surveillance. This report highlights how multiple mandibular OKCs in an 11‐year‐old boy led to the diagnosis of GS and discusses the diagnostic considerations and conservative management strategy adopted to preserve the developing dentition and support favorable craniofacial development.

## 2. Case Presentation

An 11‐year‐old boy was referred to the Dentistry Department of Sant Joan de Déu Children′s Hospital (Barcelona, Spain) for evaluation of a hard swelling affecting the right hemimandible. The patient had a history of right ocular enucleation secondary to congenital microphthalmia. Clinical examination revealed multiple punctate pigmented papular lesions located on the lateral and anterior cervical regions. The patient had previously been diagnosed with attention deficit hyperactivity disorder and was receiving methylphenidate hydrochloride (Rubicrono®).

Intraoral examination revealed early mixed dentition and moderate buccal and lingual cortical expansion in the fourth quadrant. A panoramic radiograph demonstrated a large, well‐defined radiolucent lesion involving the right mandibular parasymphyseal region, associated with displacement of the developing first and second premolars, which appeared overlapped. The right mandibular canine was positioned horizontally near the inferior border of the mandible. Additional bilateral radiolucent lesions were observed in the mandibular angle regions, causing displacement of the developing third molars (Figure 1). Cone‐beam computed tomography (CBCT) confirmed the presence of multiple well‐defined cystic lesions. Coronal and axial views demonstrated buccal and lingual cortical expansion, and the largest lesion measured 26.8 × 19.7 × 33.1 *m* *m* (Figure 1). Based on the radiographic appearance and multiplicity of the lesions, a provisional diagnosis of multiple OKCs was established.

**Figure 1:**
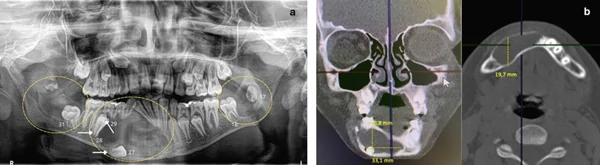
(a) Initial panoramic radiograph showing multiple cystic lesions (circles) affecting the mandible and displacement of the developing permanent teeth (arrows). Teeth are identified according to the FDI two‐digit notation system. (b) Cone‐beam computed tomography (CBCT) images in coronal and axial sections demonstrating the dimensions and cortical expansion associated with the right mandibular parasymphyseal lesion.

A conservative treatment strategy was planned to minimize morbidity and preserve the developing dentition. Under local anesthesia and moderate oral sedation with midazolam (0.3 mg/kg), the right mandibular first primary molar was extracted. Through the extraction socket, the lesion was curetted and tissue specimens were obtained for histopathological examination. Histopathological analysis revealed fragments of fibrous tissue lined by parakeratinized stratified squamous epithelium with hyperchromatic basal cells and parakeratotic superficial layers. Intraluminal keratin debris and a chronic inflammatory infiltrate within the cyst wall were also observed. These histopathological features were consistent with an OKC. A polyvinyl chloride drainage tube (5 mm in diameter and 36 mm in length) with a blind end and lateral perforations was inserted into the cystic cavity to allow continuous irrigation, drainage, and decompression. The device was secured to the attached gingiva using a 3/0 silk suture. Three weeks later, the patient underwent a second surgical procedure under general anesthesia. Bilateral cystectomy of the paramolar lesions and extraction of the mandibular third molars were performed. Curettage of the parasymphyseal lesion was repeated, and the drainage tube was replaced. Histopathological examination of the surgical specimens confirmed the diagnosis of OKCs (Figure 2).

**Figure 2:**
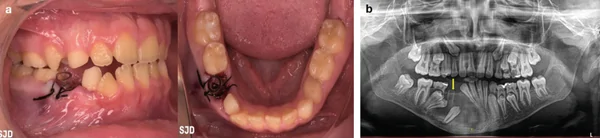
(a) Intraoral clinical photograph showing the decompression tube secured to the attached gingiva after placement in the right mandibular parasymphyseal lesion. (b) One‐month follow‐up panoramic radiograph showing maintenance of decompression, enucleation of the bilateral mandibular angle lesions, and extraction of the mandibular third molars.

At the 1‐month follow‐up visit, clinical and radiographic examination demonstrated improvement in the position of the displaced premolars and mandibular canine. A shorter drainage tube was inserted to maintain decompression; however, several weeks later, it became dislodged as a consequence of tooth eruption (Figure 3a).

**Figure 3:**
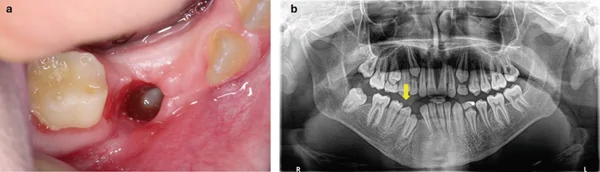
(a) Clinical image showing the opening of the decompression tract with visualization of the erupting lower right premolar. (b) Eighteen‐month follow‐up panoramic radiograph demonstrating progressive bone regeneration, reduction in lesion size, spontaneous eruption of the premolars, and favorable repositioning of the impacted canine prior to orthodontic assessment.

In addition, biopsy of the cervical papular lesions confirmed the presence of basal cell carcinoma. No other abnormalities were detected on clinical examination. In view of the presence of multiple OKCs and associated clinical findings, the patient was referred for genetic evaluation. Sanger sequencing identified a heterozygous PTCH1 variant, NM_000264.5(PTCH1):c.1340T>A (p.Leu447∗), and segregation analysis performed in both parents demonstrated that the variant had arisen de novo. According to ACMG/AMP guidelines, the variant was classified as likely pathogenic (PVS1, PM2_supporting). These findings confirmed the diagnosis of Gorlin syndrome. Family history was negative for GS, and both parents were clinically unaffected.

The patient fulfilled multiple diagnostic criteria for GS, including the major criteria of multiple OKCs, basal cell carcinoma, and a likely pathogenic de novo PTCH1 variant. Congenital microphthalmia may represent an ocular anomaly compatible with the minor diagnostic criteria of the syndrome (Table 1).

Following diagnosis, multidisciplinary assessment was undertaken, including clinical genetics evaluation, genetic counseling, dermatological assessment, and long‐term clinical and radiographic follow‐up. The previously established conservative treatment strategy was maintained to preserve the developing dentition and support favorable craniofacial development.

After 18 months of radiographic surveillance, progressive bone regeneration, reduction in lesion size, and favorable spontaneous eruption and repositioning of the affected premolars were observed. Because of the favorable positional changes of the impacted canine, the patient was referred for orthodontic assessment to determine the need for future traction (Figure 3b). Clinical monitoring was subsequently continued every 6 months for an additional year, with no evidence of recurrence after a total observation period of 30 months (Table 2).

**Table 2:** Timeline of clinical, diagnostic, therapeutic, and follow‐up events.

Timepoint	Clinical event
Initial presentation	Referral for evaluation of multiple mandibular radiolucent lesions associated with displacement of developing permanent teeth.
Baseline imaging	Panoramic radiography and CBCT revealed multiple well‐defined mandibular cystic lesions compatible with odontogenic keratocysts.
First intervention	Extraction of the right mandibular first primary molar, decompression of the largest lesion, and biopsy under local anesthesia and oral midazolam sedation.
Histopathological diagnosis	Histopathological examination confirmed odontogenic keratocyst.
Dermatological assessment	Biopsy of cervical papular lesions confirmed basal cell carcinoma.
Genetic evaluation	Sanger sequencing identified a heterozygous PTCH1 variant, NM_000264.5(PTCH1):c.1340T>A (p.Leu447∗).
Segregation analysis	Parental testing demonstrated a de novo variant. Classification: likely pathogenic (ACMG/AMP criteria PVS1, PM2_supporting).
Multidisciplinary assessment	Clinical genetics evaluation, genetic counseling, dermatological assessment, and follow‐up planning.
18 months	Panoramic radiography demonstrated progressive bone regeneration, reduction in lesion size, spontaneous eruption of premolars, and favorable repositioning of the impacted canine.
Orthodontic referral	Referral for orthodontic assessment to determine the need for future canine traction.
30 months	Total clinical follow‐up: no evidence of recurrence.

## 3. Discussion

The clinical relevance of the present case lies not only in the occurrence of multiple OKCs in a child with GS, but also in the fact that these lesions constituted the initial manifestation leading to diagnosis. OKCs are among the most characteristic manifestations of GS and constitute one of its major diagnostic criteria. In many patients, particularly during childhood and adolescence, they may represent the earliest clinically detectable feature of the disorder. Consequently, the presence of multiple OKCs in a young patient should prompt careful evaluation for an underlying syndromic condition, even when other manifestations are subtle or have not yet been recognized [7]. Early identification may facilitate timely genetic confirmation, multidisciplinary assessment, and long‐term surveillance. In the present patient, recognition of multiple mandibular OKCs prompted genetic investigation, led to the diagnosis of GS, and enabled multidisciplinary follow‐up. The initial differential diagnosis included dentigerous cysts, ameloblastoma, OKCs, and other multilocular odontogenic radiolucent lesions [8]. Histopathological examination confirmed the diagnosis of OKC.

Given the patient′s age and the presence of developing permanent teeth associated with the lesions, a conservative treatment approach was considered the most appropriate strategy. The management of OKCs remains controversial because of their locally aggressive behavior and their tendency to recur. Several therapeutic approaches have been described, including simple enucleation, enucleation combined with adjunctive therapies, marsupialization, decompression, and en bloc resection [8, 9]. Selection of the most appropriate treatment should consider lesion size, anatomical location, patient age, proximity to vital structures, and the potential impact on developing dentition.

In the present case, an individualized treatment strategy was selected for each lesion. Decompression was chosen for the largest cyst because of its dimensions and its close relationship with the developing permanent teeth. Continuous decompression reduces intracystic pressure, promotes progressive shrinkage of the lesion, and facilitates bone regeneration while minimizing the risk of damage to adjacent anatomical structures [9–11]. This approach has also been associated with preservation of tooth vitality and eruption potential, making it particularly suitable for growing patients [10, 11]. Following lesion size reduction, selective surgical treatment was performed to achieve disease control while minimizing treatment‐related morbidity. Conscious sedation with oral midazolam was used to facilitate patient cooperation and has been reported as a safe and predictable approach in pediatric dental care [12].

For the smaller lesions located in the mandibular angle regions, enucleation was performed simultaneously with extraction of the associated third molars. This approach allowed complete removal of the lesions while maintaining low surgical morbidity and favoring spontaneous osseous healing [8]. Several adjunctive therapies have been proposed to reduce recurrence following enucleation. Carnoy′s solution and modified Carnoy′s solution act by eliminating epithelial remnants and satellite cysts, whereas topical 5‐fluorouracil (5‐FU) has recently emerged as a promising alternative with fewer adverse effects and encouraging outcomes [9, 13]. However, because of the patient′s age, the proximity of the lesions to developing teeth, and the satisfactory clinical response achieved, no adjunctive therapy was considered necessary.

Recurrence rates of OKCs vary considerably, ranging from 12% to 58.3%, largely because of differences in treatment modalities, follow‐up duration, lesion characteristics, and whether syndromic lesions are included in the study population [8, 14–16]. Although simple enucleation is one of the most frequently employed techniques, several authors have reported lower recurrence rates when enucleation is preceded by decompression or marsupialization [15, 16].

Important differences have been described between sporadic and syndromic OKCs. Compared with solitary lesions, OKCs associated with GS tend to occur at a younger age, are more frequently multiple, may affect both jaws, and exhibit higher recurrence rates [17, 18]. Therefore, identification of multiple OKCs in a child should alert clinicians to the possibility of GS and prompt comprehensive clinical and genetic assessment

Long‐term radiographic surveillance is a fundamental component of patient management. The first international BCNS consensus recommended periodic panoramic radiographs during childhood and adolescence to facilitate early detection of new lesions [7]. More recent guidelines have proposed surveillance protocols tailored to age and genetic status, particularly in individuals with pathogenic PTCH1 variants [19]. Similarly, recommendations from the US National Cancer Institute emphasize balancing early lesion detection with minimization of radiation exposure [20]. Since patients with PTCH1 mutations may present increased radiosensitivity, diagnostic and follow‐up imaging should adhere to the ALARA principle (As Low As Reasonably Achievable) and nonionizing imaging modalities should be considered whenever appropriate [21, 22].

In the present patient, the combination of multiple OKCs, basal cell carcinoma, congenital ocular abnormalities, and the identification of a likely pathogenic de novo PTCH1 variant fulfilled the diagnostic criteria for GS [7]. Importantly, the odontogenic lesions were the clinical finding that prompted the diagnostic investigation and subsequent multidisciplinary assessment. This case therefore highlights the pivotal role of pediatric dentists in recognizing early manifestations of rare genetic disorders and facilitating timely diagnosis and long‐term management.

## 4. Limitations

This report is limited by its single‐case design. Although no recurrence was observed after 18 months of radiographic surveillance and 30 months of clinical follow‐up, longer observation is required given the recurrence potential of syndromic OKCs. In addition, the original histopathological photomicrographs were unavailable for inclusion.

## 5. Conclusion

Multiple OKCs in childhood should raise suspicion of GS and prompt comprehensive clinical, radiological, histopathological, and genetic evaluation. Conservative management combining decompression and selective enucleation may preserve developing dentition, support craniofacial growth, and reduce treatment‐related morbidity. Early diagnosis and long‐term multidisciplinary follow‐up are essential owing to the recurrent nature of OKCs and the multisystem involvement associated with GS. In these patients, surveillance strategies should aim not only to detect recurrence and associated manifestations but also to minimize cumulative exposure to ionizing radiation whenever possible.

## Author Contributions

V.A‐O., P.C‐B., L.B‐L., Y.G‐C., and J.M‐R. contributed to the diagnosis, treatment planning, multidisciplinary management, and follow‐up of the patient. V.A‐O., L.B‐L., E.I.M., J.M‐R. contributed to data collection, interpretation of the clinical findings, and manuscript preparation. L.B‐L. and J.M‐R. conceptualized the report and supervised the work.

## Funding

No funding was received for this manuscript.

## Disclosure

All authors critically reviewed the manuscript, approved the final version, and agreed to be accountable for all aspects of the work.

## Ethics Statement

This case report was conducted in accordance with the principles of the Declaration of Helsinki and institutional regulations governing patient confidentiality and data protection. According to institutional policies and national regulations, ethical approval was not required for the publication of this single‐patient case report

## Consent

Written informed consent was obtained from the patient′s legal guardians for publication of the clinical information and all accompanying clinical, radiographic, and genetic data included in this report

## Conflicts of Interest

The authors declare no conflicts of interest.

## Supporting information


**Supporting Information** Additional supporting information can be found online in the Supporting Information section. CARE checklist.

## Data Availability

The data supporting the findings of this case report are available from the corresponding author upon reasonable request.
